# Effect of photobiomodulation in the balance between effector and regulatory T cells in an experimental model of COPD

**DOI:** 10.3389/fmed.2024.1347517

**Published:** 2024-06-05

**Authors:** Auriléia Aparecida de Brito, Karine Zanella Herculano, Cristiano Rodrigo de Alvarenga-Nascimento, Cintia Estefano-Alves, Cinthya Cosme Gutierrez Duran, Rodrigo Labat Marcos, José Antonio Silva Junior, Maria Cristina Chavantes, Stella Regina Zamuner, Flávio Aimbire, Laia Lladó-Pelfort, Albert Gubern, Anna Fàbrega, Renata Kelly da Palma, Ana Paula Ligeiro de Oliveira

**Affiliations:** ^1^Universidade Nove de Julho, São Paulo, Brazil; ^2^Departament of Research Development and Innovation, Innovative Health System Health Management (HIS Medicine and Technology), São Paulo, Brazil; ^3^Departament of Surgery, Faculty of Veterinary, University of São Paulo, São Paulo, Brazil; ^4^Translational Medicine, Federal University of São Paulo-UNIFESP, São José dos Campos, Brazil; ^5^Department of Basic Sciences, Faculty of Health Sciences at Manresa, University of Vic-Central University of Catalonia (UVic-UCC), Manresa, Spain; ^6^Faculty of Medicine, University of Vic-Central, Manresa, Spain; ^7^Tissue Repair and Regeneration Laboratory (TR2Lab), Institute for Research and Innovation in Life and Health Sciences in Central Catalonia (Iris-CC), Vic, Spain; ^8^Faculty of Health Sciences at Manresa, University of Vic-Central University of Catalonia (UVic-UCC), Manresa, Spain; ^9^University Center of Anápolis, Anápolis, Brazil

**Keywords:** COPD, photobiomodulation (PBM), T-cells, lung, cytokines, Foxp3

## Abstract

**Introduction:**

Currently, Chronic Obstructive Pulmonary Disease (COPD) has a high impact on morbidity and mortality worldwide. The increase of CD4+, CD8+ cells expressing NF-κB, STAT4, IFN-γ and perforin are related to smoking habit, smoking history, airflow rate, obstruction and pulmonary emphysema. Furthermore, a deficiency in CD4^+^CD25^+^Foxp3^+^ regulatory T cells (Tregs) may impair the normal function of the immune system and lead to respiratory immune disease. On the other hand, the anti-inflammatory cytokine IL-10, produced by Treg cells and macrophages, inhibits the synthesis of several pro-inflammatory cytokines that are expressed in COPD. Therefore, immunotherapeutic strategies, such as Photobiomodulation (PBM), aim to regulate the levels of cytokines, chemokines and transcription factors in COPD. Consequently, the objective of this study was to evaluate CD4^+^STAT4 and CD4^+^CD25^+^Foxp3^+^ cells as well as the production of CD4^+^IFN- γ and CD4^+^CD25^+^IL-10 in the lung after PBM therapy in a COPD mice model.

**Methods:**

We induced COPD in C57BL/6 mice through an orotracheal application of cigarette smoke extract. PMB treatment was applied for the entire 7 weeks and Bronchoalveolar lavage (BAL) and lungs were collected to study production of IFN- γ and IL-10 in the lung. After the last administration with cigarette smoke extract (end of 7 weeks), 24 h later, the animals were euthanized. One-way ANOVA followed by NewmanKeuls test were used for statistical analysis with significance levels adjusted to 5% (*p* < 0.05).

**Results:**

This result showed that PBM improves COPD symptomatology, reducing the number of inflammatory cells (macrophages, neutrophils and lymphocytes), the levels of IFN-γ among others, and increased IL-10. We also observed a decrease of collagen, mucus, bronchoconstriction index, alveolar enlargement, CD4+, CD8+, CD4+STAT4+, and CD4+IFN-γ+ cells. In addition, in the treated group, we found an increase in CD4+CD25+Foxp3+ and CD4+IL-10+ T cells.

**Conclusion:**

This study suggests that PBM treatment could be applied as an immunotherapeutic strategy for COPD.

## 1 Introduction

According to WHO data (1), the main causes of human mortality, accounting for more than 68% of deaths, are chronic non-communicable diseases, many of which are related to smoking. The use of tobacco products, particularly cigarette smoke, represents the most important preventable public health problem for developed countries. These diseases include cardiovascular conditions (particularly acute myocardial infarction), cancer, stroke and chronic obstructive pulmonary disease (COPD). COPD, despite being considered a preventable and treatable condition, has become a major public health problem over the last few decades. Currently, it is among the top 3 leading causes of mortality and morbidity worldwide, with 90% of the deaths occurring in low- and middle-income countries (LMICs) (2).

The most frequent chronic symptoms in COPD include cough, sputum production, dyspnea and/or exacerbations, providing evidence of airflow impairment triggered by an abnormal inflammatory response and structural changes (2). COPD differentially encompasses several clinical phenomena. On one side, chronic bronchitis, mainly characterized by increased mucus secretion causing luminal obstruction of small airways and epithelial remodeling, among others (3). On the other side, emphysema, characterized by alveolar enlargement and alveolar wall destruction (4). Both conditions contribute to the remodeling and narrowing of small airways and destruction of the lung parenchyma. Thus, such structural changes determine that airflow limitation is largely irreversible.

COPD results from a complex not yet completely understood interaction between environmental and genetic factors over the lifetime of the individual. Regarding environmental exposure, smoking has been identified as the major determining factor in high-income countries whereas household air pollutants are considered to have a higher impact in LMICs. Air pollution is another key risk factor with global impact. Regarding the genetic traits several mutations have been described as internal risk factors (1). In fact, its pathophysiology is mainly triggered by chronic exposure to cigarette smoke and/or other irritants or pollutants, which activates a chronic inflammation state characterized by exacerbated recruitment of several types of immune cells, such as neutrophils, macrophages, CD8^+^ T (Tc1-T-helper 1 cells) and CD4^+^ T (Th1- type-1 cytotoxic T-cells and Th17) lymphocytes, into the lung parenchyma (5). Consequently, patients with COPD present increased numbers of macrophages in several clinical samples like bronchoalveolar lavage (BAL), cytology and sputum. This condition is attributed to the exacerbated recruitment of circulating monocytes in response to elevated chemokine levels, particularly those of CCL2 and CXCL1 (6). Interestingly, macrophages from these patients release more inflammatory mediators (i.e., IL-1β, IL-6, TNF-α, CXCL1, CXCL8, CCL2, LTB4) and reactive oxygen species (ROS) than those from individuals without COPD (7–9). Moreover, transcription factor STAT-4 is critical for the differentiation of Th1/Tc1 and the production of interferon (IFN)-γ. In this sense, Th1 cells and IFN-γ cytokine are increased in the airways of smokers with COPD (10). On the other hand, the anti-inflammatory cytokine IL-10, produced by Treg cells and macrophages (11), inhibits the synthesis of several pro-inflammatory cytokines that are expressed in COPD. IL-10 levels have been shown to be reduced in the sputum of COPD patients (12).

Therefore, the best pharmacologycal strategies for COPD treatment is to use anti-inflammatory compounds. Unfortunately, most currently available drugs have proven to be ineffective or characterized by unacceptable toxicity (13, 14). In the last 10 years photobiomodulation (PBM) therapy has emerged as a promising approach for the treatment of lung diseases (15). This strategy consists in the use of electromagnetic waves within the spectral range of red to near infrared (660–1,000 nm) to modulate cell functions. The outcomes depend on the administered dose, being able to inhibit or stimulate cell functions (16–20). It is noteworthy that several studies point to an anti-inflammatory role of PBM therapy on pulmonary inflammation by reducing edema, neutrophil influx, TNF-α production and increasing lung IL-10 levels (15, 21, 22). Indeed, in experimental models of COPD, data indicate the augmentation of IL-10 levels together with increased expression of this molecule in the lung after PBM (21).

The aim of this study is to enhance our understanding of PBM therapy at the molecular and cellular level, using a known experimental model of COPD, with the goal of improving management and treatment of this chronic and disabling disease.

## 2 Material and methods

### 2.1 Animals

The study was approved by the Ethics Committee on Animal Use (CEUA- AN006/2013) of Universidade Nove de Julho. Sixty healthy male C57Bl/6 mice, ~7 weeks old, weighing an average of 25.0 g, were used for the study. These specimens came from the breeding sector of the animal facility at Universidade Nove de Julho, where they were kept in good health in ventilated racks, with five animals per box, in a room with controlled environmental conditions (temperature: 22 ± 3°C, 12-h light-dark cycle and relative humidity between 30 and 70%).

### 2.2 Experimental groups

All mice were placed in a common box and divided randomly into three groups containing ten animals each: (1) control group, which consisted of non-manipulated mice; (2) PMB group, with animals exposed to PBM therapy; (3) COPD group, which consisted of animals who had been orotracheally administered cigarette smoke extract; and (4) COPD + PBM group, mice with induced COPD by cigarette extract orotracheal administration which were exposed to PBM therapy.

#### 2.2.1 Model of COPD induced by cigarette smoke extract

**Figure 1:**
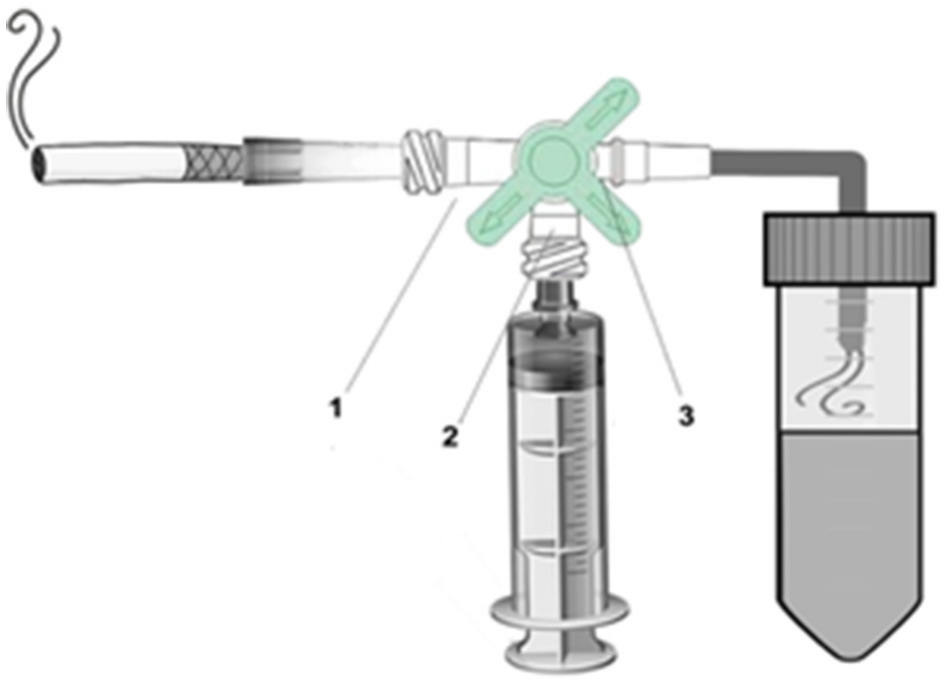
Cigarette smoke extract device. 1. Cigarette valve. 2. Syringe valve. 3. Conical tube valve.

The technique was adapted according to He et al. (23). A 20 mL plastic syringe equipped with a 3-way stopcock and a 50 mL conical tube was used to bubble smoke from 1 cigarette through 4.0 mL of deionized water (Figure 1). For disease induction, 30 μL of cigarette extract were administered orotracheally 3 times a week (Monday, Wednesday, and Friday) for 7 weeks to each animal. In order to allow access to the orotracheal administration route, animals were adequately immobilized after being anesthetized with 2% xylazine (0.06 ml/100 g) + 10% ketamine (0.08 ml/100 g).i.m. All animals belonging to experimental groups 3 (COPD) and 4 (COPD + PBM) were exposed to this protocol. After the last administration with cigarette smoke extract (end of 7 weeks), 24 h later, the animals were euthanized.

#### 2.2.2 Model of PBM-treated mice

On day 35, the animals were irradiated with a diode laser, with a power of 100 mW and a wavelength of 660 nm, irradiating an area of 0.045 cm^2^ with an energy density of 3 J. We based on the parameters of the previous study on the effect of PBM in an experimental model of asthma (22). One hour following the cigarette extract orotracheal injection, each mouse in group 4 (COPD + PBM) was given a 60-s direct application at three distinct regions: one underneath the trachea, and the other two in the right and left lung lobes, for a total of 180 s of exposure per mouse, once a day. Animal hair in this region was not removed. PBM was used for the full 7 weeks.

### 2.3 Assessment of pulmonary inflammation in bronchoalveolar lavage

Following the experiments, all groups were euthanized by giving them xylazine (10 mg/kg i.p.) and ketamine (100 mg/kg i.p.). They were also exsanguinated for blood collection, had their tracheas cannulated, had their tracheostomies, and their lungs were cleaned with 3 × 0.5 ml of phosphate buffered saline (PBS) after the extraction (24). For 5 min, the recovered lavage volume was centrifuged at 1,600 rpm and 4°C. The supernatant was kept at −70°C in preparation for ELISA cytokine analysis. The cell button was reconstituted in 1 milliliter of phosphate-buffered saline (PBS) and utilized in the production of a cytospin slide (Thermo Scientific), stained with Instant Prov (Newprov), and BAL cell determination using a Neubauer chamber (total cells). Differential cells were to do slides in cytospin and stained with Instant Prov.

### 2.4 Evaluation of cytokines, inflammatory mediators and chemokines in BAL

BAL sample collection was carried out as abovementioned. The levels of IL-1β, IL-6, KC/CXCL1, IFN-γ, TNFα, IL-10, GM-CSF, MCP-1, and LTB4 were evaluated in the BAL supernatant by ELISA, using the Biolegends and R&D kit Systems. A SpectraMax i3 (Molecular Devices) spectrophotometer with an adjusted absorbance of 450 nm was used for plate reading. The threshold for ELISA tests were: IL-1β is 31.3–2,000 pg/mL, TNF-α is 7.8–500 pg/mL, IL-6 is 7.8–500 pg/mL, IFN-γ is 31.3–2,000 pg/mL, MCP-1/CCL2 is 7.8–500 pg/mL, GM-CSF is 7.8–500 pg/mL, KC/CXCL1 is 15.6–1,000 pg/mL, LTB_4_ is 10.3–2,500 pg/mL, and IL-10 is 31.3–2,000 pg/mL.

### 2.5 Flow cytometry

Following lung extractions, lung tissue was broken up into tiny fragments and incubated for 30 min at 37°C with constant stirring in 2 mg/ml collagenase IV and 1 mg/ml deoxyribonuclease I (DNAse) (Sigma). Following this time frame, we introduced Hank's balanced solution (HBSS) together with EDTA to decelerate the material's digestion. After massing and filtering the lung fragments through a 40-mm sieve, the contents were centrifuged for 10 min at 1,500 rpm, and the pellets were then resuspended in PBS buffer. Following the incubation period, the samples were resuspended in 200 μl of the same buffer after being cleaned with PBS containing 0.01% BSA and sodium azide. The samples were acquired in the BD Accuri flow cytometer and analyzed in the CSampler software (Becton Dickinson—BD^®^, East Rutherford, NJ, USA).

Treg cell phenotyping was carried out using anti-CD4 FITC and anti-CD25 PE, as well as the transcription factor [anti-Foxp3 Percp], and IL-10 (anti-IL-10 APC) (Becton Dickinson—BD^®^, East Rutherford, NJ, USA) was characterized. The cells were incubated at 4°C for 20 min. Following the incubation period, the samples were resuspended in 200 ml of the same buffer after being cleaned with PBS containing 0.01% BSA and sodium azide. The BD Accuri flow cytometer was used to collect the samples, and the CSampler program (Becton Dickinson—BD^®^, East Rutherford, NJ, USA) was used for analysis.

The acquired cells were treated for 30 min at 4°C with a 1:100 concentration of eBioscienc^®^ anti-CD16/32 monoclonal antibody to inhibit Fc receptors in order to assess the expression of surface molecules. The cells were then treated for 30 min at 4°C with fluorochrome conjugated monoclonal antibodies (FITC, PE, PercP, or APC) specific for the compounds of interest. The following Biolegend^®^ monoclonal antibodies were used: 0.5 mg/106 cells of anti-CD3, 0.5 mg/106 cells of anti-CD4, 0.5 mg/106 cells of anti-CD25, 0.5 mg/106 cells of anti-Foxp3, and 0.5 mg/106 cells of anti-IL10. A BD Accuri flow cytometer (Becton Dickinson—BD^®^, East Rutherford, NJ, USA) was used to collect the samples. Following that, CD3+/CD4+ and CD3+/CD8+ lymphocyte populations were gated separately. Following that, STAT4+ and IFN-γ+ were gated in CD4+cells, and this population also included CD25+Foxp3+ and IL-10+. Each marker's mean fluorescence intensity (MFI) was measured and utilized in a statistical analysis. Ten mice were used in each experimental group for the sake of the results. Consequently, each sample yielded 20,000 occurrences. For the animals in the Control, COPD, and COPD + PBM groups, representative MFI histograms were acquired. One animal from each category is represented by the data.

### 2.6 Histology assessment of airway inflammation and remodeling and mucus production

In order to evaluate how different treatments affected the amount of collagen fibers in the airway wall, left lungs were removed, preserved in 10% formalin, and then embedded in paraffin. Sections of 4 μm thickness were made.

Collagen fibers were detected on slides stained with Picrosirius for the assessment of airway remodeling and inflammation. Vieira et al. (24) described a morphometric technique that was used for quantitative analysis. The NIH, Maryland, USA, Image Pro Plus software (version 4.5) was used to evaluate morphological characteristics. Every animal's five airways were examined.

Periodic acid Schiff stain was used on slides to measure mucus production. The morphometric method was followed to achieve quantification (24). With the use of Image Pro Plus software (version 4.5, NIH, Maryland, USA), morphological parameters were assessed. Each animal's five full airways were measured at a magnification of 1,000 ×. The bronchial epithelium's area of interest (mm^2^) was first determined, and the mucus area (mm^2^) was then computed:

Therefore, the unit of mucus quantification in the airways is in mm^2^/mm^2^.

### 2.7 Measurement of the mean linear intercept (Lm)

The mean linear intercept (Lm) is an index used to determine emphysema by measuring the mean diameter of the distal airspaces. Fifteen fields were counted per slide at 200X magnification. Using the reticulum superimposed on the lung parenchyma in the most peripheral regions of the parenchyma the number of times the intercepts crossed the alveolar walls were counted. The fifteen fields for each slide were averaged and the Lm was calculated using the following equation: Lm = 2,500 μm/average of the number of times the intercepts crossed the alveolar walls. The value of 2,500 μm was determined by measuring the used reticle with ruler manufactured by Zeiss. The size of each line segment was measured using the ruler under a microscope with the reticle at 200X magnification (25). The sum of all the reticulum segments resulted in the value 2,500 μm.

### 2.8 Statistical analysis

Data were analyzed using GraphPad Prism 5.1 software (California, USA). The normality of data distribution was assessed using the Kolmogorov-Smirnov test. Data with parametric distribution were submitted to the One-way ANOVA test, followed by the Newman-Keuls test for group comparison. Data with non-parametric distribution were submitted to the One-way ANOVA on Ranks test, followed by the Dunn's test for group comparison. Graphs were generated using the GraphPad Prism 5.1 software (California, USA).

## 3 Results

### 3.1 Quantification of cells present in BAL

**Figure 2:**
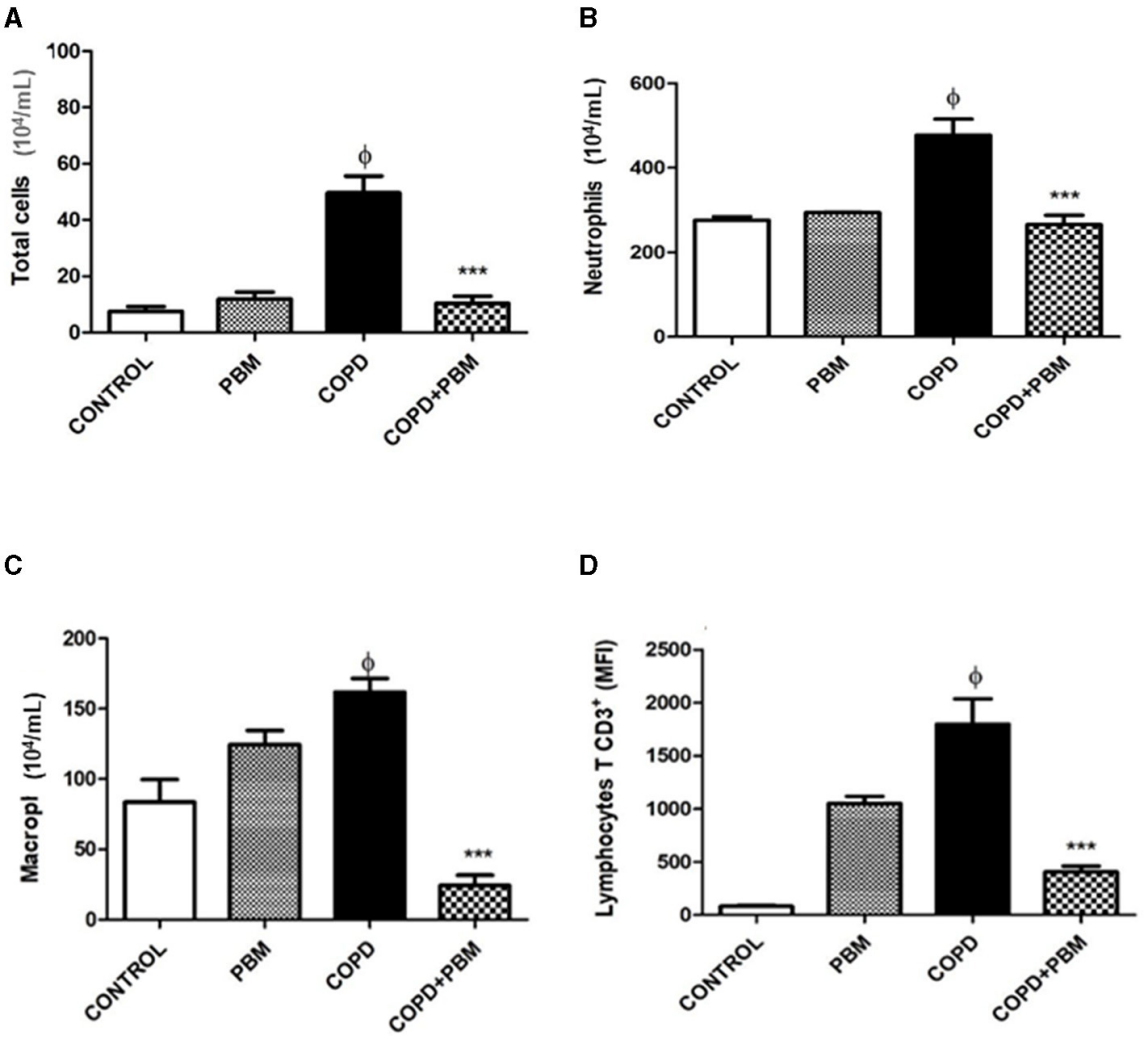
Effect of PBM with no total number of cells **(A)** and no number of neutrophils **(B)**, macrophages **(C)**, and lymphocytes **(D)** on BALF. The results refer to the use of 10 mice in each experimental group and five mice for control group. After the last administration with cigarette smoke extract (end of 7 weeks), 24 h later, the animals were euthanized. Values expressed as mean and standard deviation. ^ϕ^*p* < 0.001 when compared to the control group; ***p < 0.001 when compared to the COPD group.

The results obtained from the quantification of cells in BAL samples are represented in Figure 2. We found a significant increase in the total number of cells (A), neutrophils (B), macrophages (C) and CD3+ T lymphocytes (D) in the COPD group when compared to the control group. Contrarily, when comparing the COPD group exposed to PBM therapy (COPD + PBM) with the COPD group, we observed a significant decrease in all cell types in the former group.

### 3.2 Quantification of cytokines, inflammatory mediators and chemokines in BALF supernatant

**Figure 3:**
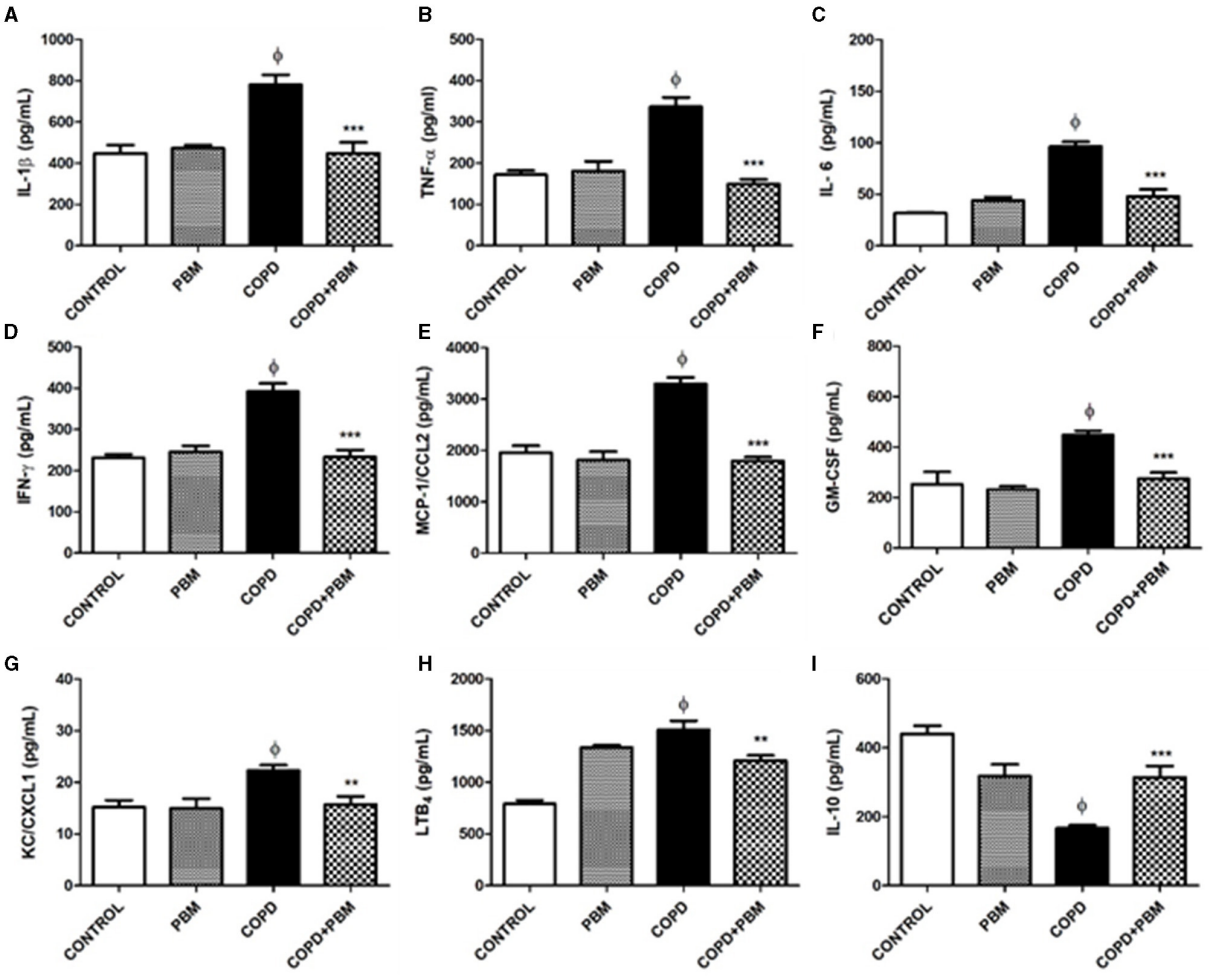
Effect of PBM on IL-1α **(A)**, TNF-β **(B)**, IL-6 **(C)**, IFN-γ **(D)**, MCP-1 **(E)**, GM-CSF **(F)**, KC/CXCL1 **(G)**, LTB4 **(H)**, and IL-10 **(I)** levels in BALF supernatant. The results refer to the use of 10 mice in each experimental group and five mice for control group. Values expressed as mean and standard deviation. ^ϕ^*p* < 0.001 when compared to the control group; *p < 0.05, **p < 0.01, and ***p < 0.001 when compared to the COPD group.

The supernatants obtained from BAL samples were used for quantification of several immune-related molecules. The results obtained are shown in Figure 3. Three different patterns may be considered. The first pattern is described regarding the levels of IL-1β, TNF-α, IL-6, IFN-γ, MCP-1/CCL2, GM-CSF, and KC/CXCL1 for which we noted a consistent and significant increase of ~2-fold in the COPD group in comparison with the control group; only in the case of KC/CXCL1 a lower although still significant increase of ~1.4-fold was observed. Moreover, in all these cases, BPM exposure of healthy mice did not lead to any significant change. In the COPD + BPM group, a significant reduction in all these molecules was observed, almost reaching control levels.

The second pattern refers to the LTB4 levels. An approximate 2-fold increase observed for the COPD-group in comparison to control animals was followed by a small but significant reduction in the COPD + BPM mice. However, we did observe that BPM exposure in healthy mice lead to LTB4 partially increased levels.

Finally, the third and contrary pattern was observed for IL-10: there was a reduction in the levels of this anti-inflammatory cytokine in the COPD group when compared with the control group, followed by a partial reversion toward basal levels. As observed for LTB4, healthy PBM-treated mice showed intermediate values.

### 3.3 Analyze of airways mucus and collagen deposition

**Figure 4:**
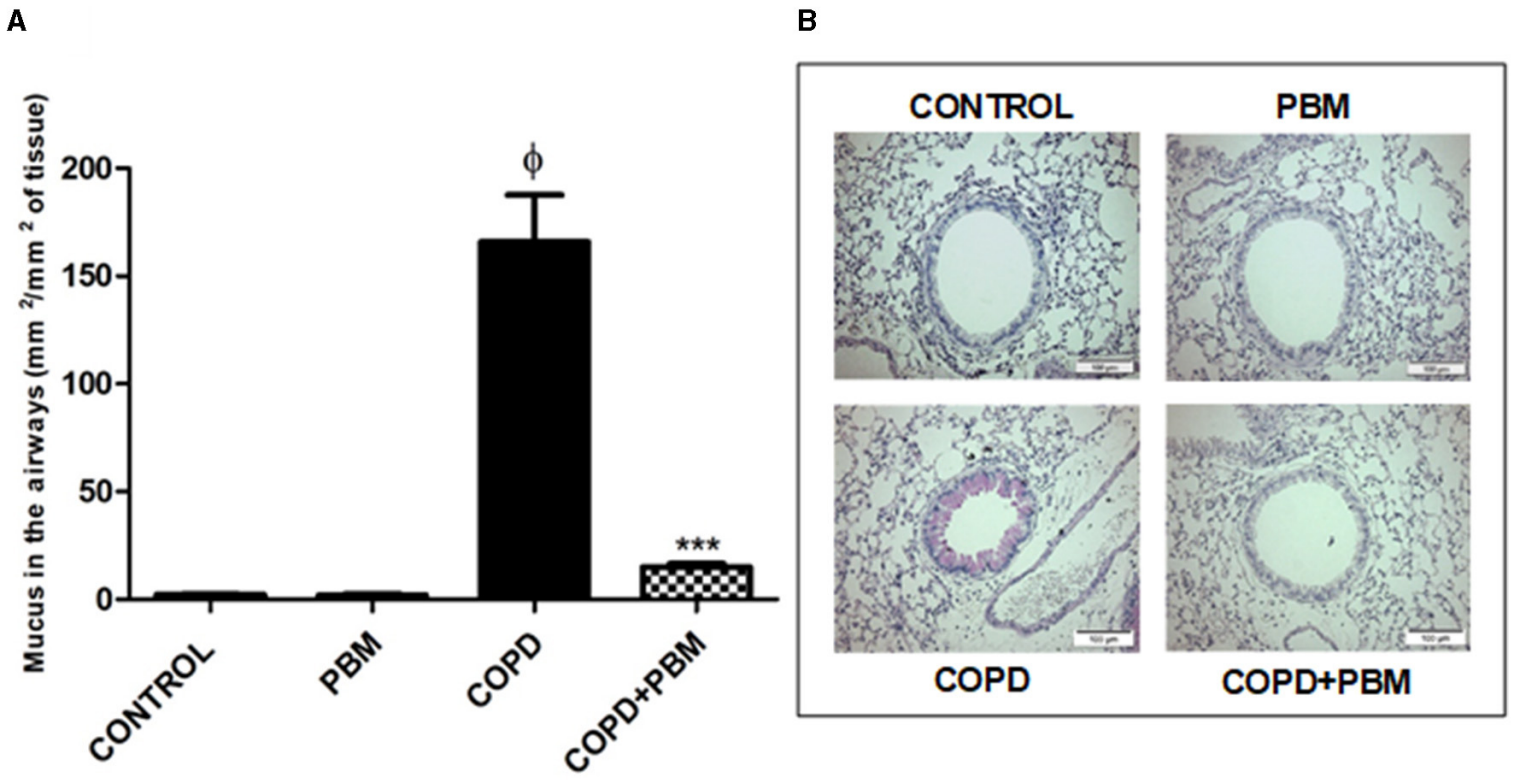
Effect of PBM on the production of mucus in the airways. **(A)**The results refer to the use of 10 mice in each experimental group and five mice for control group. Values expressed as mean and standard deviation. ^ϕ^*p* < 0.001 when compared to the control group; ***p < 0.001 when compared to the COPD group. **(B)** The photomicrographs (x 200) represent all the evaluated groups.

Regarding the production of mucus, we noticed a significant increase in the COPD group when compared to the control group. In the COPD+PBM there was a significant reduction of mucus production when compared to the COPD group (Figures 4A, B).

**Figure 5:**
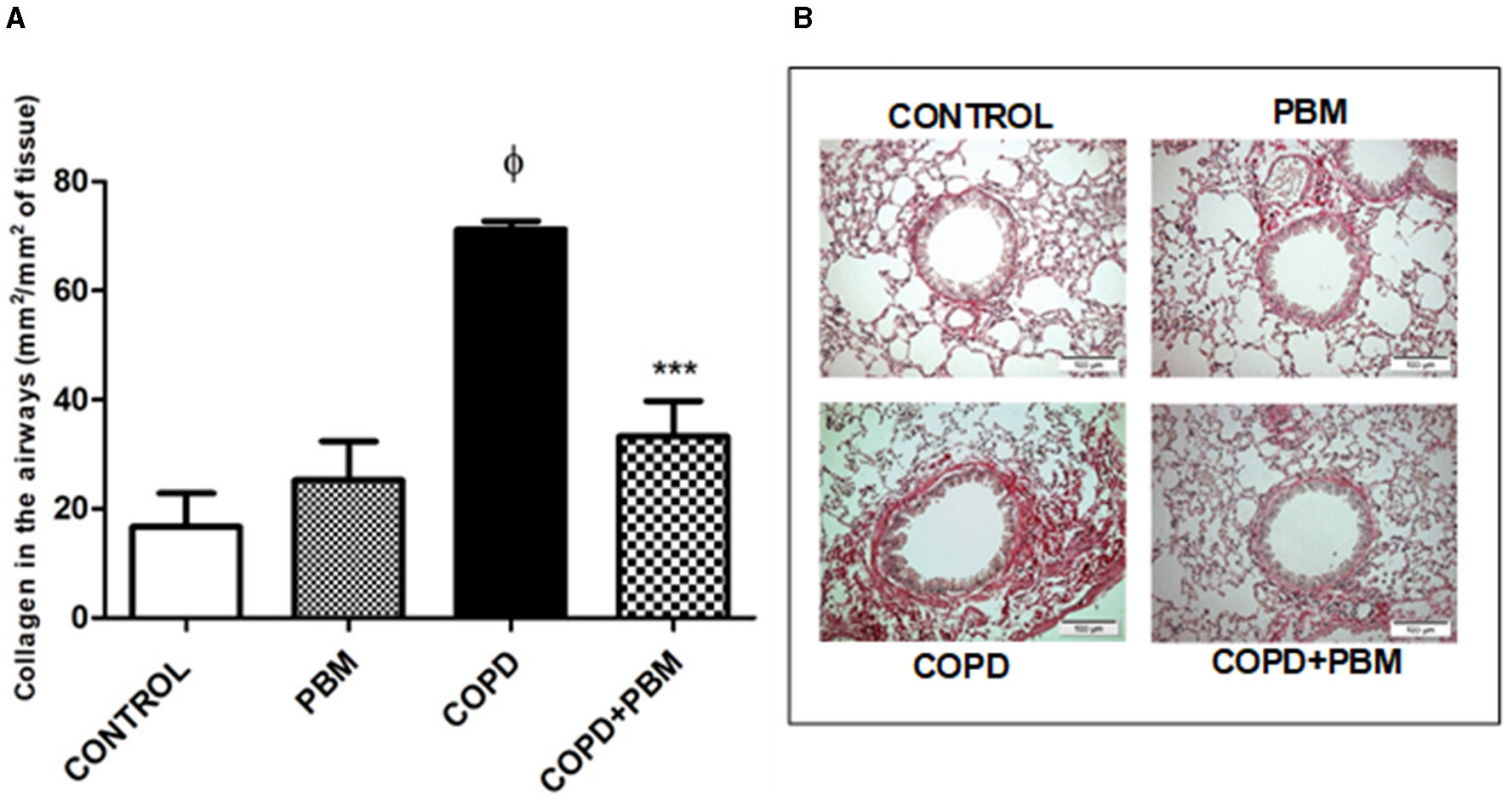
Effect of PBM on the collagen deposition in the airways. **(A)** The results refer to the use of 10 mice in each experimental group and five mice for control group. Values expressed as mean and standard deviation. ^ϕ^*p* < 0.001 when compared to the control group; ***p < 0.001 when compared to the COPD group. **(B)** The photomicrographs (x 200) represent all the evaluated groups.

The collagen deposition also showed an increase in the COPD group compared to the control group and a significant reduction in COPD + PBM group when compared to COPD (Figures 5A, B).

### 3.4 Emphysema determination

**Figure 6:**
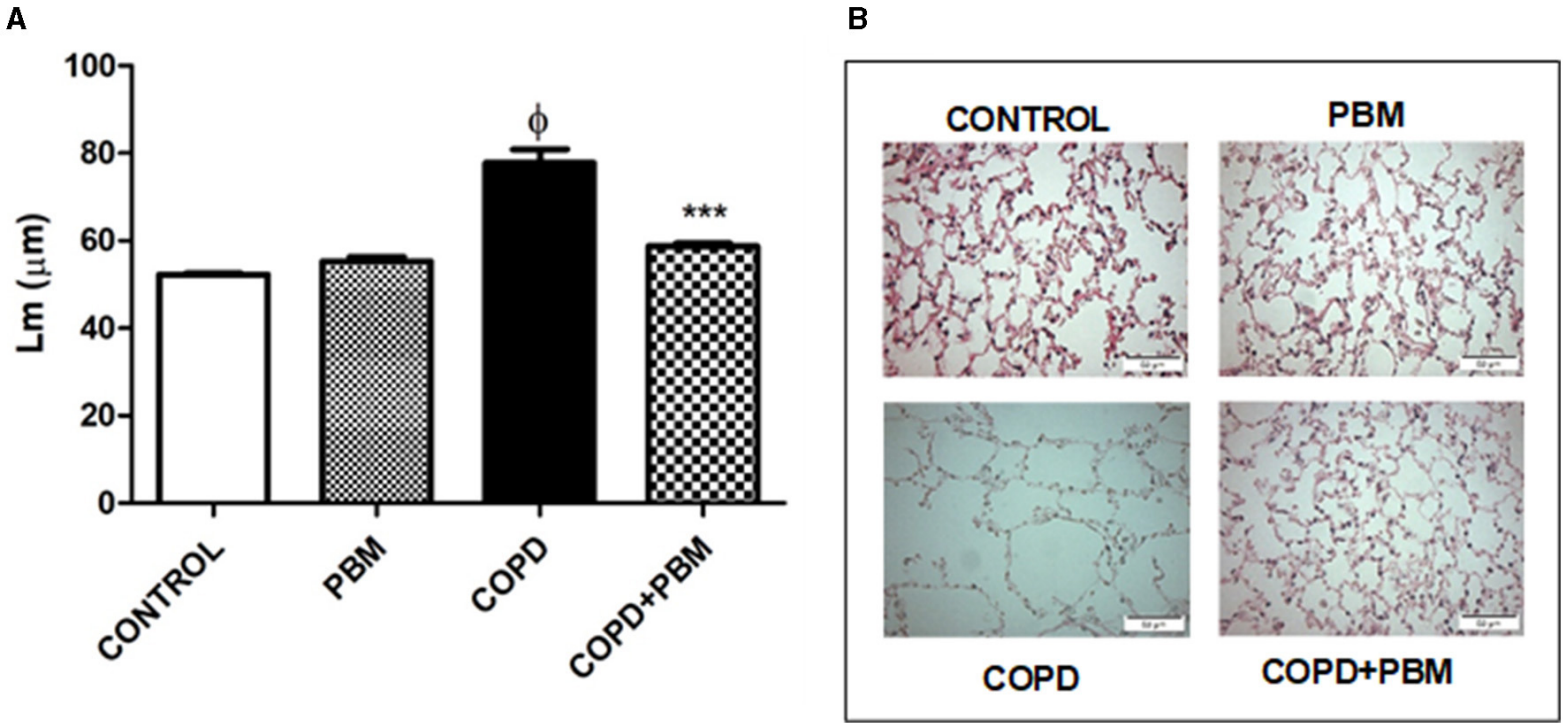
Effect of PBM on the alveolar enlargement in the airways. **(A)** The results refer to the use of 10 mice in each experimental group and five mice for control group. Values expressed as mean and standard deviation. ^ϕ^*p* < 0.001 when compared to the control group; ***p < 0.001 when compared to the COPD group. **(B)** The photomicrographs (x 200) represent all the evaluated groups.

Alveolar enlargement data showed a significant increase in the COPD group when compared to the control group. Contrarily, a significant reduction was detected in the COPD + PBM group (Figure 6) compared to the COPD group.

The bronchoconstriction index showed an increase in the COPD group compared to the control group and a significant reduction in the COPD + PBM group (Figure 6).

### 3.5 Quantification of CD4+ and CD8+ T lymphocytes in BAL

**Figure 7:**
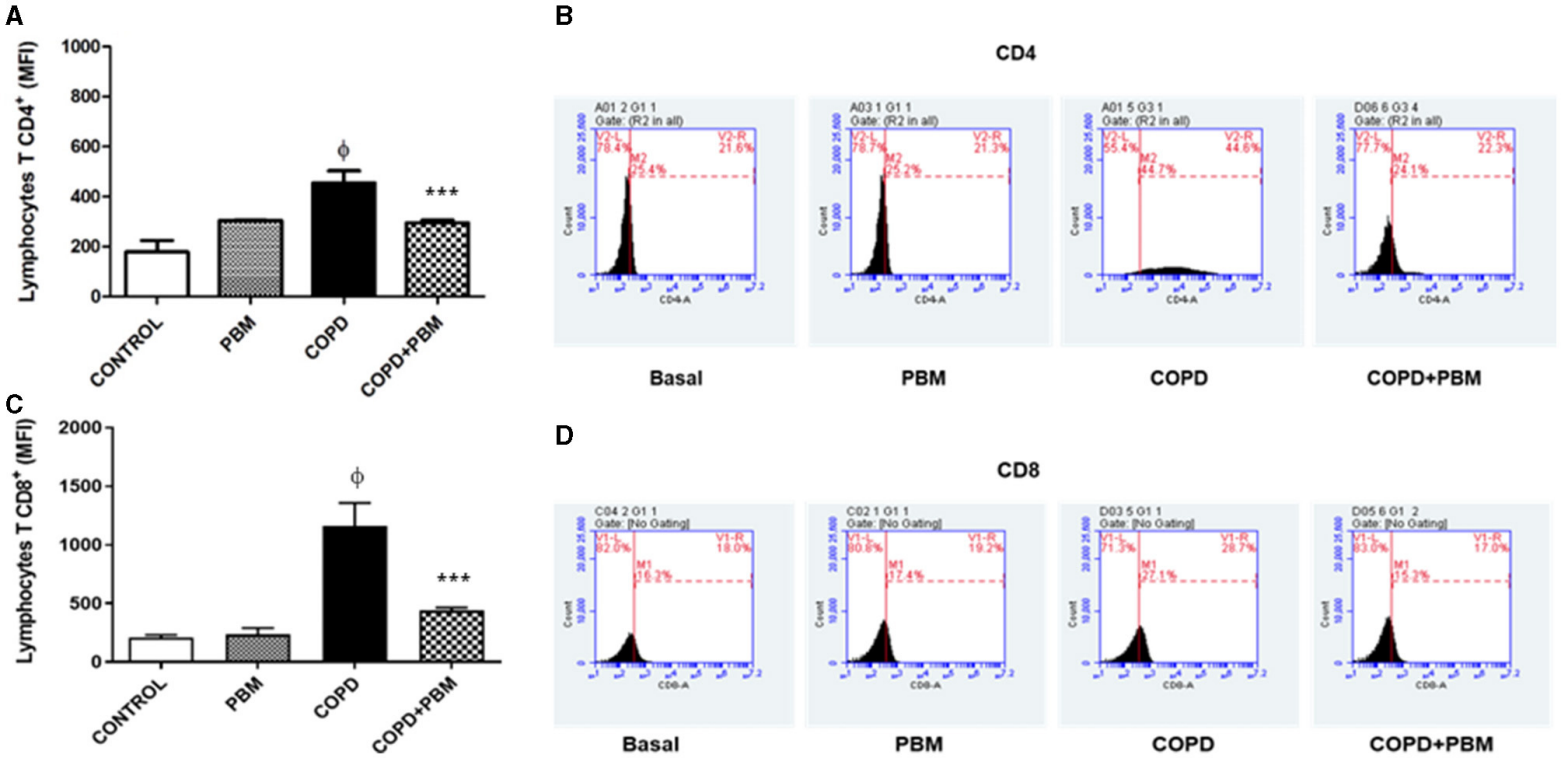
Effect of PBM therapy on lymphocytes T CD4+ **(A)** and lymphocytes T CD8+ **(C)** levels in BALF. The results refer to the use of 10 mice in each experimental group and 5 mice for control group. Values expressed as mean and standard deviation. The histograms represent all groups evaluated **(B, D)**. Values expressed as mean and standard deviation. ^ϕ^*p* < 0.001 when compared to the control group; ***p < 0.001 when compared to the COPD group.

The results of the quantification of CD4+ (A) and CD8+ (B) lymphocytes in BAL demonstrated a significant increase in the COPD group compared to the control group (Figure 7). On the other hand, the COPD+PBM group had a reduction in CD4+ and CD8+ when compared to the COPD group.

### 3.6 Quantification of CD4+STAT-4+ and CD4+STAT-4+IFN-γ+ T lymphocytes in BAL

**Figure 8:**
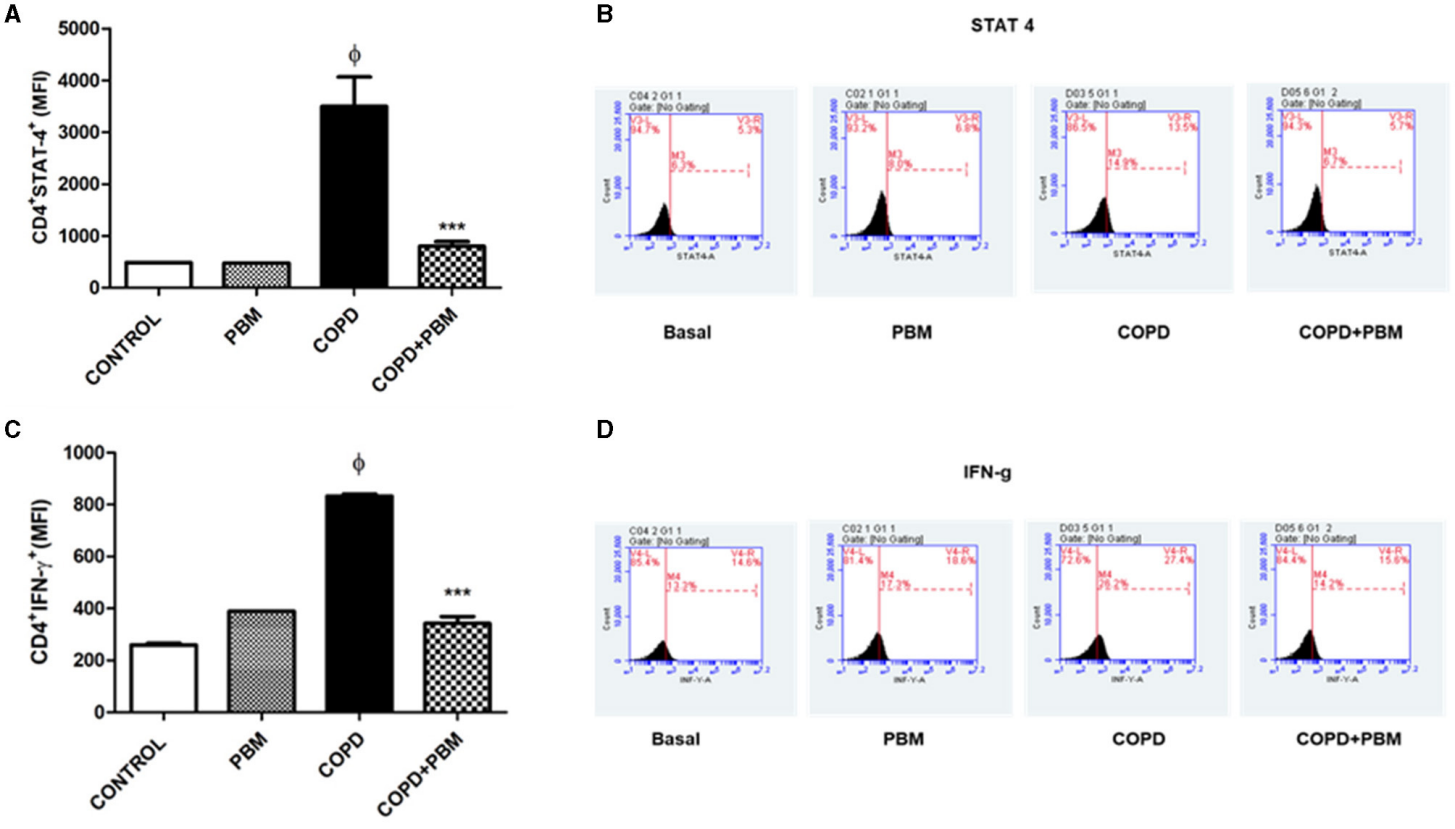
Effect of PBM therapy on CD4+IFN-y + **(A)** and CD4+STAT-4 +**(C)** levels in BALF. The results refer to the use of 10 mice in each experimental group and five mice for control group. Values expressed as mean and standard deviation. The histograms represent all groups evaluated **(B, D)**. Values expressed as mean and standard deviation. ^ϕ^*p* < 0.001 when compared to the control group; ***p < 0.001 when compared to the COPD group.

The results of CD4+STAT-4+ lymphocytes (Figure 8A) and CD4+IFN-γ+ (Figure 8B) in BAL showed a significant increase in the COPD group compared to the Basal group and a significant reduction in the PBM treated group. The histogram represents all groups evaluated (Figures 8C, D).

### 3.7 Quantification of CD4+CD25+Foxp3+ and CD4+CD25+IL10+ T lymphocytes in BAL

**Figure 9:**
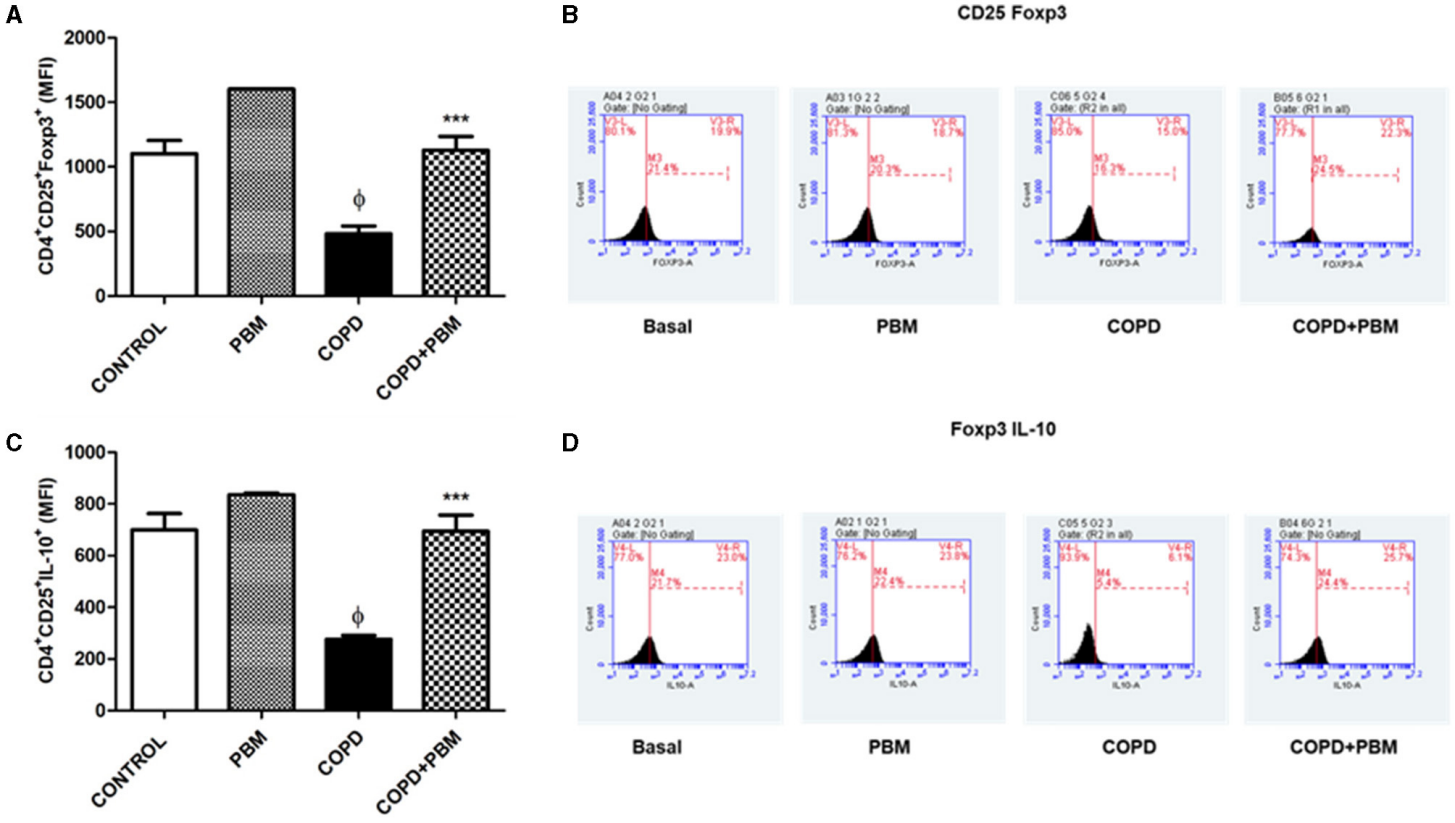
Effect of PBM therapy on CD4+CD25+FOXP3+ **(A)** and CD4+CD25+ IL-10+ **(C)** levels in BALF. The results refer to the use of 10 mice in each experimental group and five mice for control group. Values expressed as mean and standard deviation. The histograms represent all groups evaluated **(B, D)**. Values expressed as mean and standard deviation. ^ϕ^*p* < 0.001 when compared to the control group; ***p < 0.001 when compared to the COPD group.

Data regarding the quantification of CD4+CD25+Foxp3+ (Figure 9A) and CD4+CD25+IL10+ (Figure 9B) T lymphocytes in BAL showed a significant reduction in the COPD group compared to the basal group and an increase in the PBM treated group. The histogram represents all groups evaluated (Figures 9C, D).

## 4 Discussion

The results obtained in the present study demonstrate that treatment with PBM irradiation significantly reduces chronic symptoms which typically emerge in a mouse COPD model induced by orotracheal administration of cigarette smoke extract. The respiratory impairment observed in COPD affected organisms mainly include small-airway remodeling and narrowing as well as pulmonary emphysema. The latter condition refers to the observed pulmonary tissue destruction, particularly at the alveolar level (4, 26). Many of the parameters evaluated in this study, such as cellularity, secretion of pro-inflammatory cytokines, expression of the inflammatory transcription factor STAT4, were significantly reduced in BAL samples collected from COPD mice treated with PBM in comparison with the control and healthy group. On the other hand, the anti-inflammatory transcription factor Foxp3 as well as IL-10 levels were locally increased. This profile was associated with better maintenance of tissue integrity when compared to untreated COPD animals, evidenced by reduced mucus secretion, collagen deposition, and tissue damage. It is worth mentioning that these characteristics have been proven to be associated with the mentioned tissue destruction and further decline in the patient's quality of life (26). In this regard, billions of dollars are spent each year on treating emphysematous patients around the world. Therefore, the need for a more efficient and cheaper therapeutic approach in the management of patients with COPD is of paramount importance.

Previous data have shown the benefits of a PBM therapy used in the mouse model of experimental COPD induced by cigarette smoke. One study showed that PBM therapy was able to reduce the levels of inflammation, airway remodeling and pulmonary emphysema, with the participation of the purinergic pathway (21). Another study previously carried out used PBM together with mesenchymal stem cells derived from human fallopian tubes. There was a reduction in both lung inflammation and alveolar enlargement in sick animals treated with PBM. Many of the parameters evaluated, such as cellularity, secretion of proinflammatory cytokines, perivascular infiltrate and expression of transcription factors such as NF-KB were significantly reduced. Both treatments, PBM in combination with mesenchymal stem cells or PBM alone, were shown to maintain the integrity of the lung tissue, reduce collagen deposition as well as the peribronchial infiltrate (27).

In this context, we evaluated the presence of inflammatory cells in BAL samples. Overall, the data obtained indicated an increase in the number of macrophages, neutrophils and lymphocytes in the samples collected from animals with COPD. Diverse studies conducted elsewhere have also evidenced increased levels of all these cell types and others in COPD patients, highlighting that increased numbers of neutrophils and B lymphocytes are detected in the most severe cases. Moreover, macrophages are of great importance in this pathogenesis and are increased not only in individuals with COPD but also in smokers (4, 5).

Our results corroborate previous findings that demonstrate a reduction in the expression of STAT4, as well as in the concentrations of IL-8 and LTB4 in the lung, which are primarily responsible for attracting neutrophils (23). This suggests that the anti-inflammatory effect of laser irradiation on these mediators, and consequently the reduction in cell migration to the lung, could be attributed to the decreased expression of STAT4 and the inhibition of IL-8 and LTB4.

Exposure to cigarette smoke initially leads to a cellular infiltrate of blood-derived neutrophils and monocytes secreting pro-inflammatory and pro-fibrotic cytokines, such as IL-1β, IL-6, IL-12, TNF-α, and TGF-β, together with chemokines, lipid mediators and several other molecules. This scenario is accompanied by degradation of the extracellular matrix by MMP-1 (matrix metalloproteinase-1), secreted by alveolar macrophages, causing tissue destruction and emphysema (28). Chronic exposure, on the other hand, leads to an intense infiltrate of CD4 and CD8 T lymphocytes, and IFN-γ is probably the most important cytokine derived from T cells. We noticed that, in animals with COPD induced by cigarette smoke extract, INF-γ-producing Th1 cells, as well as those expressing STAT4, were increased, whereas Treg cells (CD4^+^CD25^+^Foxp3^+^) were decreased.

Furthermore, granulocyte macrophage colony-stimulating factor (GM-CSF) has been connected to COPD and is involved in the differentiation and survival of neutrophils, eosinophils, and macrophages (29). In reaction to inflammatory stimuli, macrophages, epithelial cells, and T lymphocytes release GM-CSF primarily. Additionally, patients with COPD secrete GM-CSF from their alveolar macrophages, which may be crucial for improving neutrophil and macrophage survival in the airways (29). Increased neutrophil counts in the lung are correlated with elevated GM-CSF concentrations in the BAL of COPD patients, particularly during exacerbations.

In this study, we observed a significant increase in the number of CD4+ T lymphocytes in the COPD groups. Conversely, the treated groups exhibited a decrease in CD4+ lymphocytes across all treatment cohorts. Other studies have reported that functional Treg cells suppressed the proliferation of activated CD4+ T cells (30), and their generation is primarily induced by the presence of TGF-β and IL-10 (31, 32). IL-10 inhibits the synthesis of numerous inflammatory proteins, including TNF-α, IL-1β, GM-CSF, chemokines, and metalloproteinases like MMP-9, which are highly expressed in COPD. Concentrations of IL-10 are reduced in the sputum of COPD patients (29).

In this context, therapeutic approaches capable of reducing this activation, could significantly contribute to maintaining pulmonary integrity. These findings suggest that PBM increased IL-10 levels produced by Treg cells in the treated group, thereby suppressing the proliferation of CD4+ T effector cells. Moreover, prior studies have indicated an inverse correlation between CD8+ T cells and lung function (33). Smoking directly disrupts the balance of the pro-/anti-inflammatory response by inhibiting CD8+ Treg cells (33). Nonetheless, further analysis of these cells is required to determine their regulatory functions or any potential relationship between cytotoxic CD8+ T cells and IL-10 production.

After analyzing these results, we propose that photobiomodulation holds the potential to reduce disease severity by upregulating IL-10 release. Several studies indicate a potential mechanism for the modulatory effects of PBM in the lungs through the release of IL-10 (34–36). Therefore, we urge other research groups to delve into the potential of laser therapy, given its promising outcomes in COPD. This underscores the necessity for further experiments, such as quantifying MMP-12 and MMP-9, characterizing the types of collagen present in lung parenchyma, and analyzing levels of IL-17, PGE2, TGF-β, ATP, and oxidative stress. Additionally, verifying the profile of regulatory CD8+ T cells is crucial, given their significant role in the pathophysiology of COPD. Furthermore, treatment given to the microbial component of COPD has not been evaluated, additional studies need to be carried out to discuss this aspect of the disease.

## 5 Conclusion

Our findings demonstrate that photobiomodulation therapy effectively reduces cell migration to the lung, levels of cytokines and chemokines, and alveolar enlargement. This reduction could potentially be attributed to the increased population of regulatory T cells (CD4+CD25+Foxp3+) producing IL-10 within the lung. Under this results, we suggest that these cells act by suppressing effector T cells (CD4+STAT-4+), known for producing IFN-γ. The significance of photobiomodulation therapy lies in its potential to regulate inflammation and treat pulmonary emphysema in individuals with COPD.

## Data availability statement

The raw data supporting the conclusions of this article will be made available by the authors, without undue reservation.

## Ethics statement

The animal study was approved by the animal study was reviewed and approved by Nove de Julho University. The study was conducted in accordance with the local legislation and institutional requirements.

## Author contributions

AB: Methodology, Conceptualization, Formal analysis, Writing – original draft. KH: Methodology, Writing – review & editing. CA-N: Writing – review & editing. CE-A: Writing – review & editing. CD: Writing – review & editing. RM: Writing – review & editing, Supervision. JS: Writing – review & editing. MC: Writing – review & editing, Resources. SZ: Writing – review & editing, Data curation. FA: Writing – review & editing, Formal analysis, Methodology, Supervision. LL-P: Writing – review & editing, Writing – original draft. AG: Writing – review & editing. AF: Writing – review & editing. RP: Methodology, Writing – review & editing. AL: Conceptualization, Data curation, Funding acquisition, Supervision, Writing – review & editing.
